# Paraglottic and Pre-epiglottic Space Abscess in Adults: Report of Two Cases

**DOI:** 10.22038/ijorl.2020.42184.2375

**Published:** 2020-05

**Authors:** Giorgos Sideris, Marilia Sapountzi, Pavlos Maragoudakis, Alexander Delides

**Affiliations:** 1 *Department of Otolaryngology, "Attikon" University Hospital, Athens, Greece. Rimini 1, Chaidari, Athens 124 62, Greece*

**Keywords:** Abscess, Airway obstruction, Epiglottitis, Supraglottitis, Tracheostomy

## Abstract

**Introduction:**

Acute epiglottitis or supraglottitis is a rapidly progressing upper respiratory tract infection that can often threaten the airway patency. Epiglottic abscess that expands to the paraglottic (PGS) or preepiglottic (PES) space and acute airway obstruction constitute rare complications, exclusively presented in adults.

**Case Report:**

We report two cases. In the first case flexible fiberoptic Rhino-Pharyngo-Laryngoscopy showed epiglottitis that was obstructing the airway and abscesses on the lingual surface of the epiglottis. Abscesses were opened using laser CO2. In the following days flexible fiberoptic endoscopy revealed persisting protrusion of the left hemilarynx. A CT scan was performed showing an abscess in the paraglottic space. Under direct laryngoscopy the abscess was drained. In the second case endoscopic examination revealed epiglottitis that did not cause airway obstruction. The patient was admitted for follow-up and treated with intravenous antibiotics. On the 5th day showed an exacerbation of her symptoms. A CT scan was performed that showed the existence of an abscess in the preepiglottic space. She was taken to surgery and the abscesses were drained through a cervical- U shaped- incission.

**Conclusion:**

Existance of an abscess means, by default, an adequate surgical treatment to ensure the airway, and immediate drainage under direct laryngoscopy or through an external approach. Diagnosis is based exclusively on medical history and clinical examination. CT scan is necessary to reveal “secret” abscesses and “silent” extension of the infection inside pre-epiglottic and paraglottic space even if epiglottitis is mild. Postoperative management includes proper care of the surgical wound and antibiotics.

## Introduction

Acute epiglottitis or supraglottitis is a rapidly progressing upper respiratory tract infection that can often threaten the airway patency.

Besides the epiglottis, it can affect the supraglottic structures such as arytenoid folds, the vestibular folds (false vocal cords) and the vallecula epiglottica. Epiglottic abscess that expands to the paraglottic and preepiglottic space, parapharyngeal abscess, Lemierre Syndrome, pneumonia and acute airway obstruction constitute rare complications, exclusively presented in adults (1)_._

## Case Report

**Fig 1:**
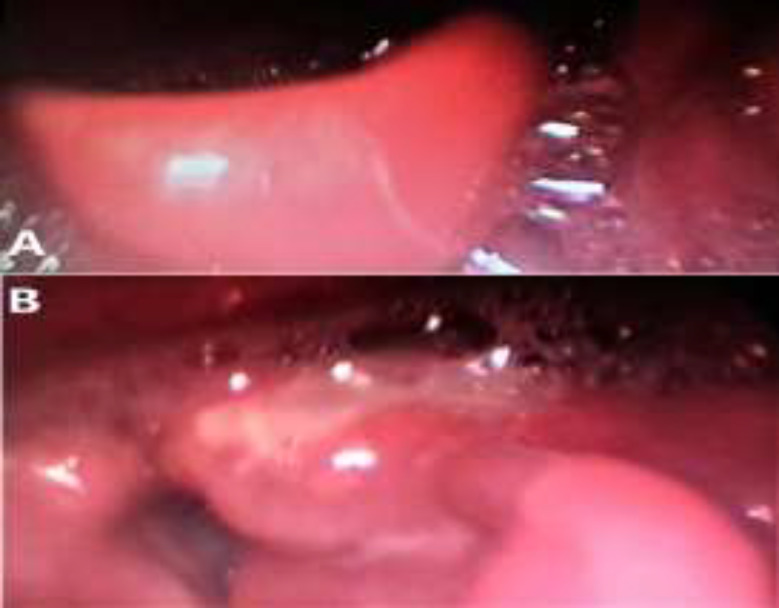
The appearance of the epiglottitis: A) thickened, edematous, reddish obstructing the airway Β) with abscesses on the lingual surface, the area over the left arytenoid fold and the right epiglottic vallecula

A 72-year old diabetic male presented at the ENT emergency department with pharyngalgia, odynophagia and fever up to 39^o^C. Clinical examination revealed a febrile patient with hypoxia, hoarseness and drooling. Flexible fiberoptic Rhino-Pharyngo-Laryngoscopy showed pooling of secretions in the pyriform sinuses, as well as a thickened, edematous, reddish epiglottis that was obstructing the airway (Fig.1).

Lab tests revealed: White Blood cell Count (WBC): 14830 Κ/μL, neutrophils: 83.4%, C-reactive protein (CRP): 101 mg/L, Glucose: 246 mg/dL. An endotracheal intubation was not considered safe and, therefore, an emergency tracheostomy was performed under local anaesthesia. Then general anaesthesia was administered followed by direct laryngoscopy that revealed abscesses on the lingual surface of the epiglottis, the area over the left arytenoid and the right epiglottic vallecula. Abscesses were opened using laser CO_2. _

**Fig 2:**
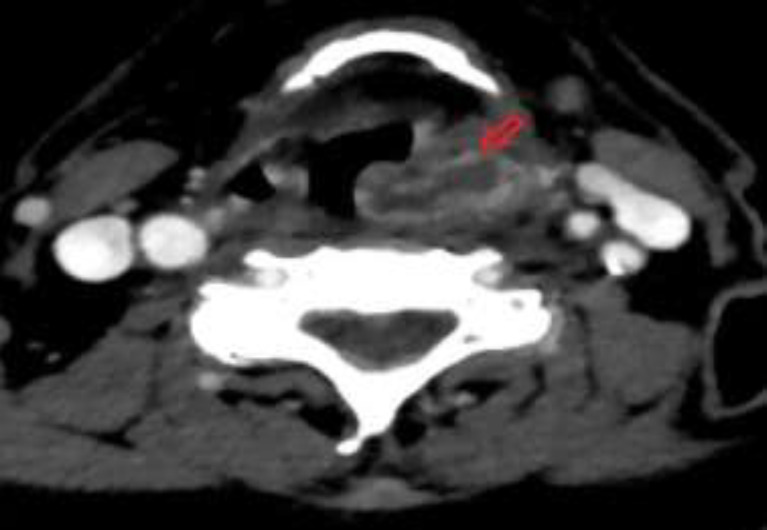
Contrast enhanced Computerized Tomography Scan of patient 1 showing an abscess in the left paraglottic space (3,1 X 0,9 X 0,5) cm

Culture were positive for Streptococcus parasanguinis (classified as member of the Streptococcus viridans group-Group A hemolytic streptococci) was isolated. The patient was awakened and moved to the ward. He was administered with ceftriaxone plus clindamycin. In the following days flexible fiberoptic endoscopy revealed persisting protrusion of the left hemilarynx. A CT scan was performed showing an abscess in the paraglottic space (3.1Χ0.9Χ0.5cm) (Fig. 2).

**Fig 3:**
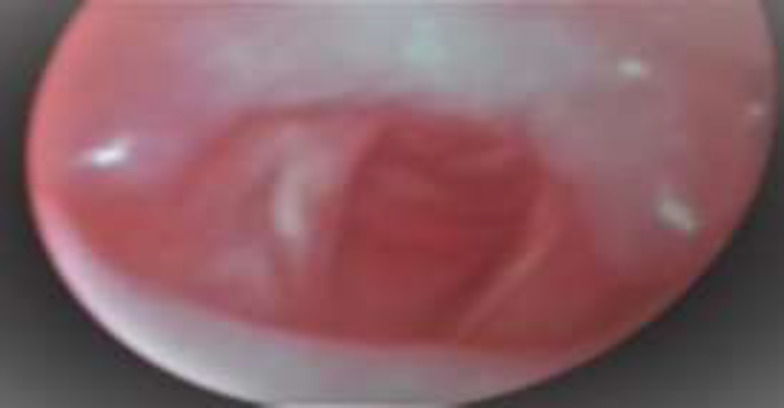
Normal larynx as observed on the13th post-operative day

He was again taken to the operating room where under general anesthesia and direct laryngoscopy the abscess was drained. He improved the following days, was decannulated on the 13^th^ post-operative day and discharged (Fig.3).

Case Report

A 41-year-old female with a free past medical history presented to the emergency department complaining of pharyngalgia, odynophagia and fever up to 39^o^C. She was hoarse and drooling. 

Endoscopic examination revealed epiglottitis that did not cause airway obstruction.

Lab tests taken during the patient’s admission showed: WBC: 15380 Κ/μL, neutrophils: 83.4%, CRP: 29.1 mg/L.

The patient was admitted for follow-up and was treated with intravenous ceftriaxone plus clindamycin.

She remain stable but on the 5^th^ day showed an exacerbation of her symptoms.

Endoscopy revealed a symmetrical edematous reddish epiglottis and involvement of both the arytenoid and false vocal folds.

**Fig 4:**
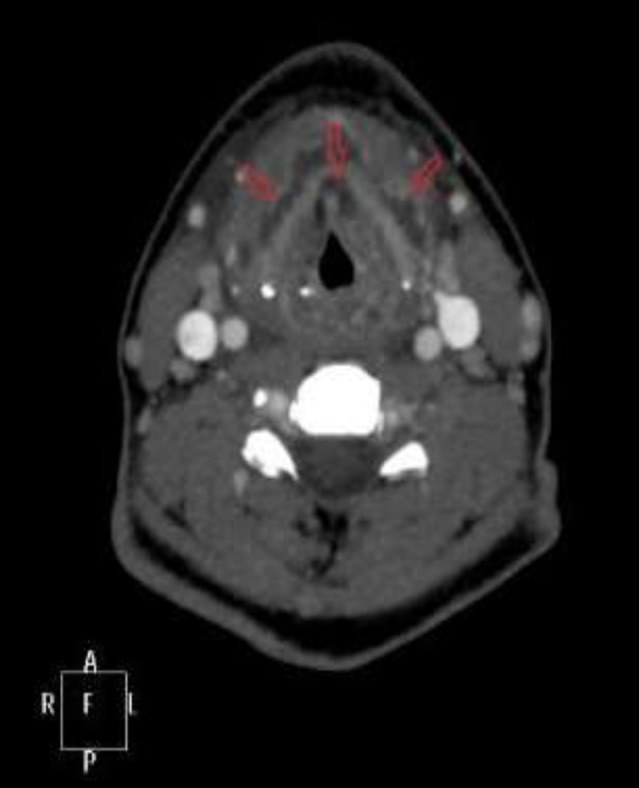
Contrast enhanced Computerized Tomography Scan of patient 2 with existence of an abscess in the preepiglottic space and small abscesses anteriorly to the thyroid cartilage (5,7 X 1,7 X 0,7) cm

A CT scan was performed that showed the existence of an abscess in the preepiglottic space with small abscesses anteriorly to the thyroid cartilage (5.7 x 1.7 x 0.7 cm) (Fig.4).

She was taken to surgery and the abscesses were drained under general anaesthesia through a cervical- U shaped- incision. 

The spaces between the superficial layer of the deep cervical fascia and the pretracheal cervical fascia were drained. 

Debridement was performed and pus as well as tissue were sent for culture. Necrotic tissue and encrustation were extensively surgically removed. Tissue cultures were positive for streptococcus agalactiae (Group B hemolytic streptococci).

**Fig 5:**
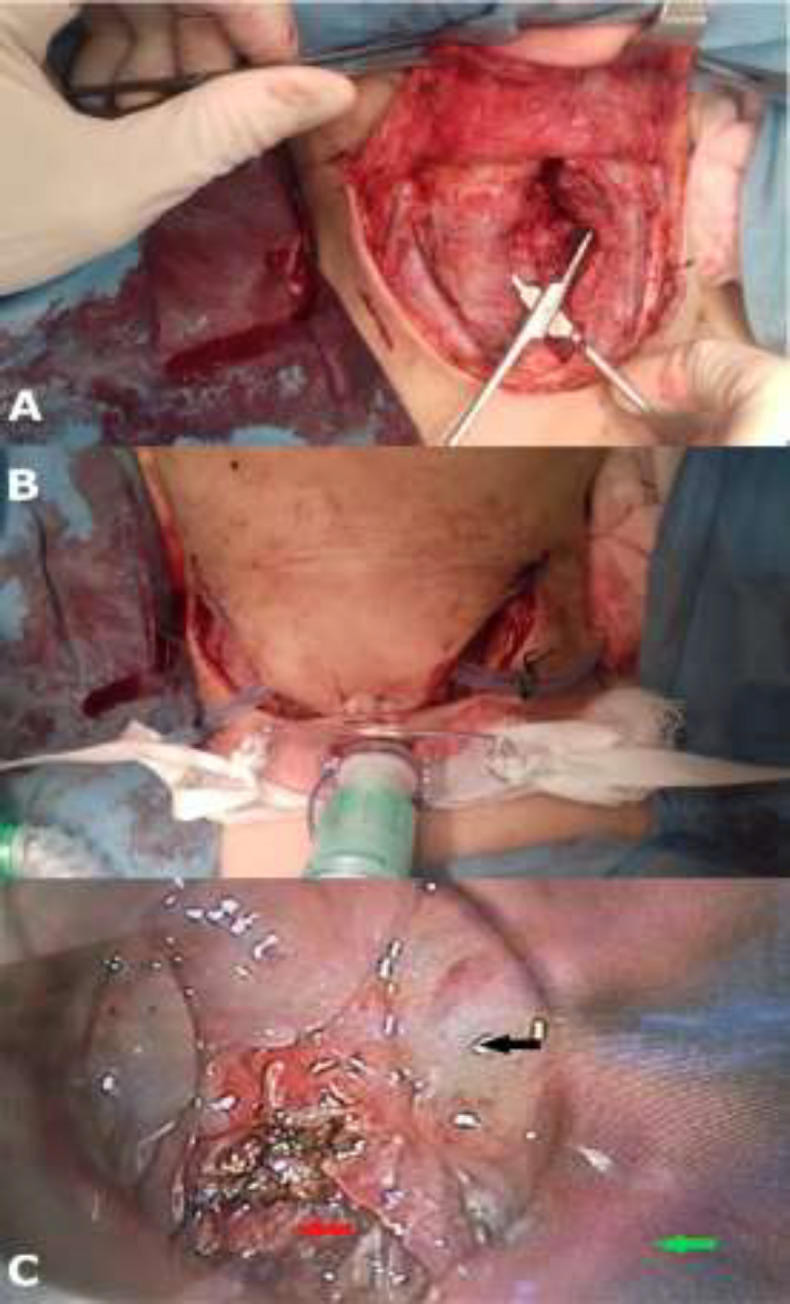
The surgical strategy: A) drainage of the spaces between the superficial layer of the deep cervical fascia and the pretracheal cervical fascia through a cervical- U shaped- incision B) tracheostomy to secure the airway C) direct (green arrow: suprahyoid epiglottis, black arrow: valeccula, red arrow: pre-epiglottic space)

A tracheostomy was then performed to secure the airway following by a direct laryngoscopy where a small quantity of pus was drained from the preepiglottic space through vallecula epiglottica (Fig.5).

The same antibiotic treatment was continued while the trauma was daily irrigated with natural saline through small hunts- “penrose” type. The patient was decannulated on the 6^th^ post-operative day and discharged on the 10^th^ post-op day.

## Discussion

Lately the number of reported cases of acute epiglottitis in adults has risen with controversial the incidence of mortality (2,3).

The disease onset is usually longer than 24 hours and flexible fiberoptic Rhino-Pharyngo- Laryngoscopy is essential for the diagnosis and reveals if the epiglottis is swollen, bulging and reddish, as the participation of supraglottic areas (4). 

Conservative measures include oxygenation, hydration and intravenous antibiotics. Use of corticosteroids is controversial.

According to the literature conservative airway management is often successful without any surgical intervention and the need of tracheostomy is rare (5,6).

However, some studies show that securing the airway by performing an emergency endotracheal intubation, tracheostomy or cricothyrotomy is not as rare as estimated. In a study by Berger et al., 25 out of the 118 patients with acute epiglottitis needed an intervention to secure the airway, immediate up to the first 2 days of hospitalization (21%) (7). 

In adults, some relatively useful clinical predicting factors of airway obstruction appear to exist. Studies show that patients who present to the emergency department in less than 8 hours of the onset of pharyngalgia (4), patients that present drooling due to severe odynophagia (4,6,7) and patients with tachypnea, stridor, hypoxia and fever have a higher risk of airway obstruction (6).

In an analysis of 6.072 cases of acute epiglottis within 599 hospitals of Japan, Suzuki et al report that high mortality or need for intubation risk factors are advanced age, body mass index >25 kg/m^2^, male sex, diabetes mellitus, epiglottic cyst, pneumonia and hospitalization in a university hospital (8). In an analysis of 308 cases in Finland, Bizaki et all report also hypertension, depression and asthma (6).

It has been shown that in the post Haemophilus inﬂuenzae type-b vaccine era the most probable cause of infection in adults is not Hib (1,2,6). This is confirmed by the two clinical cases we present in which streptococcus was isolated.

Bacterial cultures of abscess or necrotic residues should be obtained “lege artis” and the results should define the choice of the antibiotic treatment (9). 

An abscess is found most often on the lingual surface of the epiglottis, a factor thought to be due to the loose mucosal covering of the cartilage in this area and the higher incidence of mucoceles on the lingual aspect (10). 

The clinical significance of the pre-epiglottic (PES) and paraglottic space (PGS) in relation to the spread of carcinoma has been known for many years. The lymphatics of the supraglottic larynx drain to cervical lymph nodes via the PES. In regards to the PGS various anatomical barriers such as the conus elasticus and the quadrangular membrane have been well documented. Both our cases discussed in this report demonstrate that acute infection of the supraglottic larynx may also spread inferiorly to involve the PES and the PGS (11,12). A few cases are reported to the international literature (13-15).

It is obvious that in severe cases, treatment should not be delayed for imaging and the patient should be transferred immediately in the operating room in order to secure the airway with intubation or tracheostomy under local anaesthesia.

CT should, of course, be considered only in patients with a stable airway, as the supine position required for CT increases the risk of acute respiratory distress. 

That raises the question: is a CT necessary in cases that don’t pose an imminent threat to the patency of the airway? In the 2 clinical cases reported in this paper, performing a CT scan revealed abscesses that could not be revealed with a fiberoptic laryngoscopy and helped planning the surgical treatment (13). Therefore, delayed detection of an abscess might be the result of an absent CT scan. 

We thus propose that routine CT in patients with acute supraglottitis even if the swelling of the epiglottitis is mild, could prevent delayed diagnosis and facilitate earlier treatment (14,16,17).

## Conclusion

Existence of an abscess means, by default, an adequate surgical treatment to ensure the airway, and immediate drainage of the abscess under direct laryngoscopy or through an external approach, by using laser or cold steel methods. Diagnosis is based exclusively on medical history and clinical examination. 

CT scan is necessary to reveal “secret” abscesses and “silent” extension of the epiglottitis inside deep neck spaces such as pre-epiglottic and paraglottic spaces even if epiglottitis is mild and of course to define the surgical plan. Postoperative management includes proper care of the surgical wound and antibiotic coverage. 
